# *bmp-2* Gene-Transferred Skeletal Muscles with Needle-Type Electrodes as Efficient and Reliable Biomaterials for Bone Regeneration

**DOI:** 10.3390/ma17040880

**Published:** 2024-02-14

**Authors:** Mariko Yamamoto Kawai, Takeshi Yoshida, Tomoki Kato, Takuma Watanabe, Marina Kashiwagi, Shigeki Yamanaka, Hiromitsu Yamamoto, Shigeki Nagahiro, Tsutomu Iwamoto, Khan Masud, Kazuhiro Aoki, Kiyoshi Ohura, Kazumasa Nakao

**Affiliations:** 1 Department of Welfare, Kansai Women’s College, Osaka 582-0026, Japan; 2 Department of Oral and Maxillofacial Surgery, Graduate School of Medicine, Kyoto University, Kyoto 606-8507, Japan; t_yoshida91@kuhp.kyoto-u.ac.jp (T.Y.); t.kato.clpc@gmail.com (T.K.); takuma@kuhp.kyoto-u.ac.jp (T.W.); kashiwagim@kuhp.kyoto-u.ac.jp (M.K.); yama0821@kuhp.kyoto-u.ac.jp (S.Y.); hyamamotokuhp@mac.com (H.Y.); knakao@kuhp.kyoto-u.ac.jp (K.N.); 3 Department of Pediatric Dentistry/Special Needs Dentistry, Division of Oral Health Sciences, Graduate School of Medical and Dental Sciences, Tokyo Medical and Dental University, Tokyo 113-8549, Japan; shigeki.nagahiro@gmail.com (S.N.); iwamoto.dohs@tmd.ac.jp (T.I.); 4 Department of Basic Oral Health Engineering, Graduate School of Medical and Dental Sciences, Tokyo Medical and Dental University, Tokyo 113-8549, Japan; mas-bhoe@tmd.ac.jp (K.M.); aok-dphm@tmd.ac.jp (K.A.); 5 Department of Nursing, Taisei Gakuin University, Osaka 587-8555, Japan; k-ooura@tgu.ac.jp; 6 Graduate School, Division of Dental Research, Osaka Dental University, Osaka 573-1121, Japan

**Keywords:** muscle, bone, *bmp-2*

## Abstract

Background: Bone morphogenetic protein-2 (*bmp-2*) has a high potential to induce bone tissue formation in skeletal muscles. We developed a bone induction system in skeletal muscles using the *bmp-2* gene through in vivo electroporation. Natural bone tissues with skeletal muscles can be considered potential candidates for biomaterials. However, our previous system using plate-type electrodes did not achieve a 100% success rate in inducing bone tissues in skeletal muscles. In this study, we aimed to enhance the efficiency of bone tissue formation in skeletal muscles by using a non-viral *bmp-2* gene expression plasmid vector (pCAGGS-*bmp-2*) and needle-type electrodes. Methods: We injected the *bmp-2* gene with pCAGGS-*bmp-2* into the skeletal muscles of rats’ legs and immediately placed needle-type electrodes there. Skeletal tissues were then observed on the 21st day after gene transfer using soft X-ray and histological analyses. Results: The use of needle-type electrodes resulted in a 100% success rate in inducing bone tissues in skeletal muscles. In contrast, the plate-type electrodes only exhibited a 33% success rate. Thus, needle-type electrodes can be more efficient and reliable for transferring the *bmp-2* gene to skeletal muscles, making them potential biomaterials for repairing bone defects.

## 1. Introduction

Recombinant human bone morphogenetic protein (rhBMP)-2 has a high potential for osteoinduction [1]. In previous studies and clinical applications, absorbable and biocompatible carrier components have been used as biomaterials to retain rhBMP-2 at the target site to achieve successful osteoinductive treatments [1,2,3,4,5,6,7,8]. The rhBMP-2 concentration required for effective osteoinduction ranges from 0.75 to 1.5 mg/mL in clinical trials [9,10]. Long-term high-dose rhBMP-2 treatment leads to osteoclastogenesis via a negative feedback mechanism [11]. Therefore, we developed a gene therapy using a viral or non-viral *bmp-2* gene expression plasmid vector for bone regeneration [12,13,14]. However, adenoviral vectors induce an immune response against transduced cells [15,16]. The expression of the human *bmp-2* gene using a viral vector induces bone tissue formation, which occurs only when immune responses are either locally or generally suppressed [15,16]. In our next strategy for safer *bmp-2* gene therapy for bone regeneration, we constructed a non-viral *bmp-2* gene expression plasmid vector. Although non-viral plasmid vectors are safer than viral vectors, their transfection efficiency is usually lower [17,18,19]. Consequently, strategies are needed to enhance the efficiency of gene transfer to cells using non-viral vectors. Non-viral approaches for gene delivery to cells can be broadly categorized into physical penetration methods, such as electroporation, gene gun, and laser techniques, as well as chemical carriers, including lipofection, lipoplexes, exosomes, synthetic nanoparticles, etc. [20,21,22]. Moreover, combinations of these methods have been developed, such as photothermal nanomaterial-mediated photoporation [23]. In our first trial, we attempted to increase the transfection efficiency of the gene using a combination of a non-viral *bmp-2* gene expression plasmid vector and in vivo electroporation [12]. We successfully induced ectopic bone formation in skeletal muscles using plate-type electrodes but did not achieve a 100% success rate [12].

**Figure 1:**
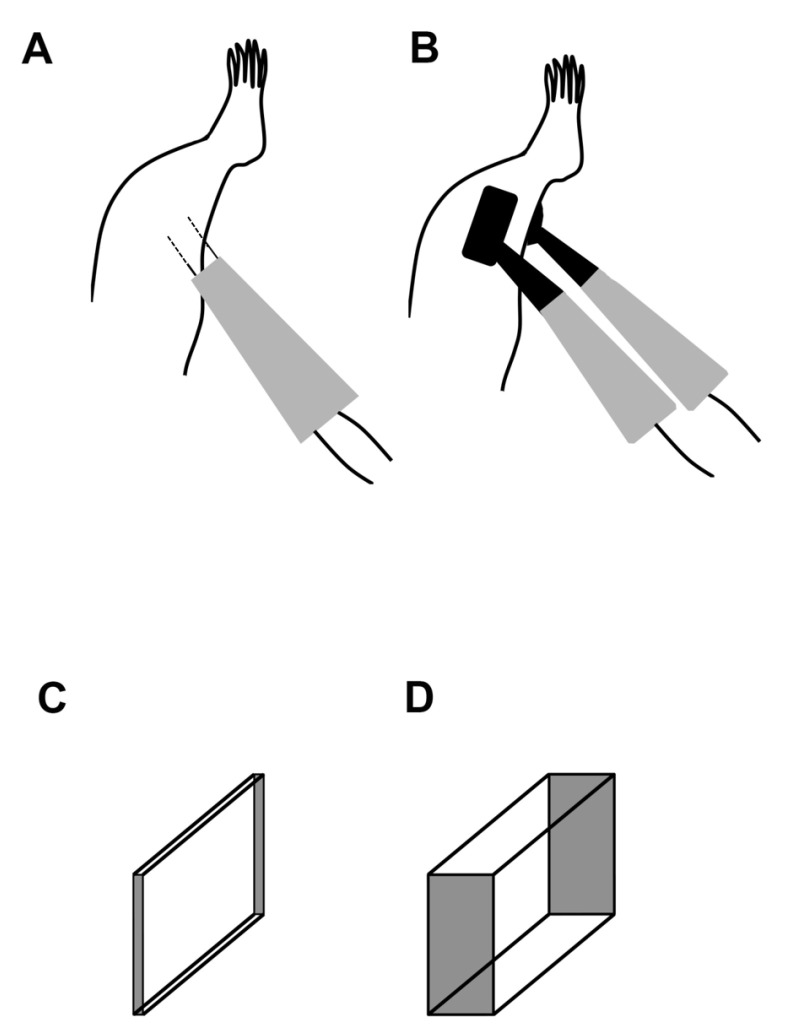
Schematic of the in vivo electroporation setup. (**A**) Needle-type electrodes were inserted directly into the targeted muscles (dotted line). (**B**) Plate-type electrodes were attached to the skin of the targeted muscles. The electrical ranges of (**C**) needle- and (**D**) plate-type electrodes are two- and three-dimensional, respectively.

It is known that several parameters affect the efficiency of gene transfer by electroporation [24,25,26]. Electric field distribution, which is one of the parameters, is related to the electrode configuration, as well as electric pulse parameters [27,28]. In the case of gene transfer to the skeletal muscles of rats, electric field distribution is visualized, such as needle-type (Figure 1C) or plate-type (Figure 1D) electrodes. Nonetheless, the electric field distribution with needle-type electrodes is less than that with plate-type electrodes; it cannot be obstructed by the skin, mucosa, and muscle fascia. This is attributed to the ability of needle-type electrodes to reach deep into target areas easily [29,30].

Bone graft with muscle could be expected to be a very promising surgery, especially intractable bone fractures, such as for femoral neck fractures, which are notorious for complications like avascular necrosis and nonunion [31,32]. In previous reports, *bmp-2* gene-transferred skeletal muscles with an adenoviral plasmid vector were used to repair large segmental bone defects in rats [33,34]. *bmp-2* gene-activated muscle grafts with adenoviral vectors exhibit bone volume and stability similar to bone isografts, mimicking autologous bone grafting in rats [34]. Compared with bone autografts, muscle tissues can be harvested in larger quantities, causing only low donor site morbidity [34,35,36,37,38]. Once gene transfer to muscle grafts has been optimized, gene-transferred skeletal muscles may become a more potent material for grafting, with higher osteoinductivity and the ability to promote faster and more robust bone defect healing. Therefore, safe and reliable *bmp-2* gene-transferred skeletal muscles could be used as biomaterials for repairing bone defects.

In this study, we aimed to enhance the efficiency of bone tissue formation in skeletal muscles by using a non-viral *bmp-2* gene expression plasmid vector and needle-type electrodes.

## 2. Materials and Methods

### 2.1. Preparation of bmp-2-Expression Plasmid

The pCAGGS-*bmp-2* plasmid expressing the human *bmp-2* gene was constructed as previously described [12]. pCAGGS-*bmp-2* contains the CAG (cytomegalovirus immediate–early enhancer/chicken β-actin hybrid) and human *bmp-2* cDNA. The plasmid was transformed into *Escherichia coli* (DH5α) and isolated using a Qiagen EndoFree Plasmid Giga Kit (Qiagen, Hilden, Germany). The DNA was diluted in phosphate-buffered saline (PBS) to a concentration of 0.5 µg/µL before injection into the rat’s skeletal muscles.

### 2.2. Gene Transfer by Electroporation with Needle-Type Electrodes

Nine-week-old male Wistar rats (n = 6 for each treatment group) were anesthetized via intraperitoneal injection of sodium pentobarbital (5 mg/100 g body weight) and the fur on the target leg area was removed with clippers. The plasmid vector was diluted with PBS to a concentration of 0.5 µg/µL and 25 µL was injected into the middle portion of each gastrocnemius muscle using a syringe with a 31-gauge needle. Needle-type electrodes made of stainless steel (Ohta Seiko Co., Okayama, Japan) with a length of 10 mm, separated by 5 mm, were inserted into the areas previously injected with the plasmid vector (Figure 1A). The surface area of the plate-type electrodes made of stainless steel (Ohta Seiko Co., Okayama, Japan) was 10 mm × 15 mm (Figure 1B). Electroporation was immediately performed using eight electrical pulses at 100 V for 50 ms with an electroporator (Ohta Seiko Co., Okayama, Japan). All animal experiments were approved by the Animal Research Control Committee of Osaka Dental University (approval no. 19-02016).

### 2.3. Radiographic Analysis

The rats were sacrificed with an overdose of sodium pentobarbital 21 days after *bmp-2* gene transfer. The injected regions of the gastrocnemius muscles were dissected and calcified tissues were detected on soft X-ray films using SRO-M50 (Sofron Inc., Tokyo, Japan). These images were captured under specific imaging conditions, with a secondary voltage of 28 kV, a current of 2.5 mA, and an exposure time of 30 s.

### 2.4. Image Analysis

We identified radiopaque areas that contained particles using soft X-ray films, plotted the perimeter of each particle-containing opacity, and measured each area using ImageJ software (access date 15 October 2021) [37]. Measurements were performed five times, and the mean values were calculated.

### 2.5. Histological Analysis

The rats were euthanized with an overdose of sodium pentobarbital 1 day or 21 days after *bmp-2* gene transfer. The dissected gastrocnemius muscles were fixed with 0.05 M phosphate-buffered 4% paraformaldehyde (pH 7.4) and embedded in paraffin. The skeletal muscles with plate-type electrodes were decalcified, cut into 7 µm thick sections, and stained with hematoxylin and eosin (HE). The skeletal muscles with needle-type electrodes were cut without decalcification and stained with HE.

### 2.6. Statistical Analyses

All of the data are presented as mean ± standard deviation. Statistical significance between the two groups was determined using an unpaired *t*-test. If a *p*-value from the *t*-test, conducted at a 95% confidence interval, was 5% or less, it was considered indicative of a significant difference between the two groups. Multiple group comparisons were performed using one-way ANOVA and Tukey’s multiple comparison post hoc tests.

## 3. Results

### 3.1. Radiographic Findings

**Figure 2:**
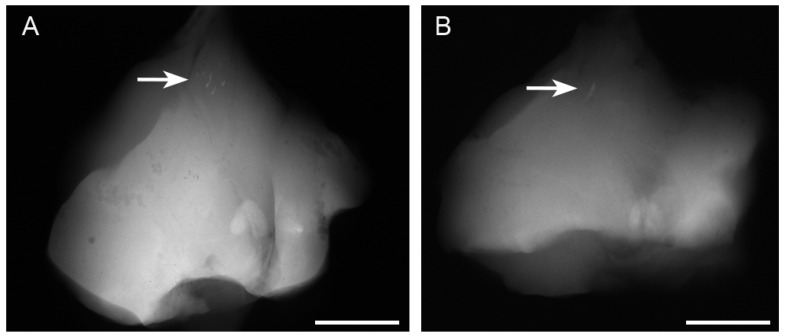
Presence of opacities on soft X-ray films after *bmp-2*-expressing plasmid electroporation. The opacities (white arrow) in the targeted muscles after electroporation with (**A**) needle- and (**B**) plate-type electrodes. Several opacities (white arrow) in the skeletal muscle *bmp-2* gene were transferred with needle-type electrodes (**A**). One opacity (white arrow) was found in the skeletal muscles with plate-type electrodes (**B**). Each scale bar is 15 mm.

**Figure 3:**
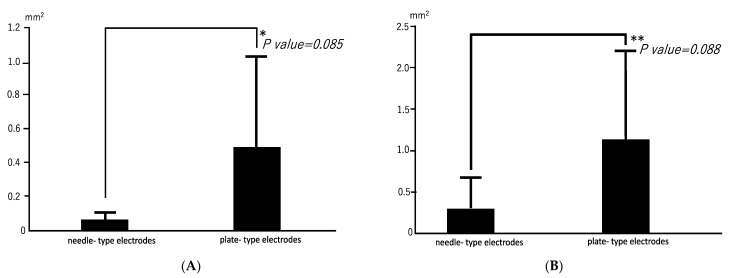
Mean areas of opacities in electroporated skeletal muscles. (**A**) Mean area for each opacity. * *p* = 0.085; 95% confidence interval: 0.2556–0.1063. (**B**) Mean total area for opacities. ** *p* = 0.088; 95% confidence interval: 0.750–0.2279.

Radiographs revealed opacities comprising several small calcified particles with clear margins in the muscles targeted for *bmp-2* transfection via in vivo electroporation using needle-type electrodes (Figure 2A). The opacities were detected in all six rats (100%). In vivo electroporation using plate-type electrodes resulted in large opacities with clear margins in the targeted muscles (Figure 2B) in two out of six rats (33%). The average areas of each particle-containing opacity were 0.071 ± 0.048 mm^2^ (needle-type electrode) and 0.494 ± 0.54 mm^2^ (plate-type electrode) (Figure 3A). The average areas of total particle-containing opacities were 0.31 ± 0.376 mm^2^ (needle-type electrode) and 1.135 ± 1.082 mm^2^ (plate-type electrode) (Figure 3B).

### 3.2. Histological Findings

**Figure 4:**
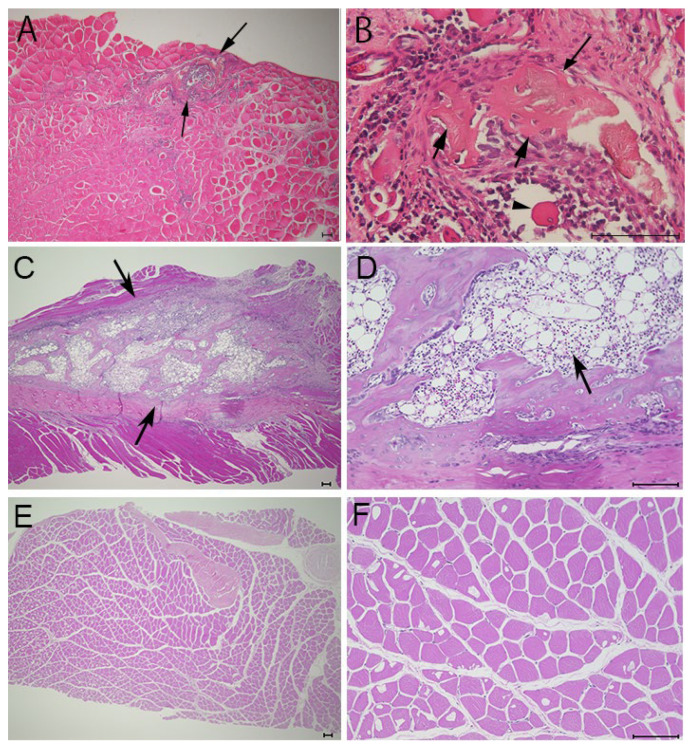
HE staining of targeted skeletal muscles 21 days after gene transfer. HE staining of muscles after electroporation with needle-type electrodes revealed the formation of several bone regions ((**A**,**B**), arrows). HE staining of muscles after electroporation with plate-type electrodes showed the formation of larger bone regions ((**C**), arrows) and lipid-rich tissues and hematocytes ((**D**), arrow). (**E**,**F**) HE staining of the muscles after electroporation with empty plasmid did not reveal the formation of any bone regions. Scale bars represent 100 µm.

To verify whether the opacities detected on soft X-ray films represented ectopic bone formation, we examined muscle sections harvested 21 days after electroporation using HE staining. Several small new bone calcifications were observed in the muscles targeted with the needle-type electrodes (Figure 4A,B, arrows). The ectopic bone tissues (Figure 4B, arrows) were stained slightly lighter with eosin than the muscle tissues (Figure 4B, arrow head) and contained cells resembling osteocytes. These small bone regions were interspersed, similar to the opacities observed on soft X-ray films (Figure 2A). In contrast, the formation of bone calcifications was larger in muscles targeted with plate-type electrodes than in those induced using needle-type electrodes. Moreover, the muscles targeted with plate-type electrodes contained bone-marrow-like tissues, including lipid-rich tissues and hematocytes (Figure 4D, arrow). Muscles targeted with empty plasmids did not exhibit ectopic bone formation (Figure 4E,F).

**Figure 5:**
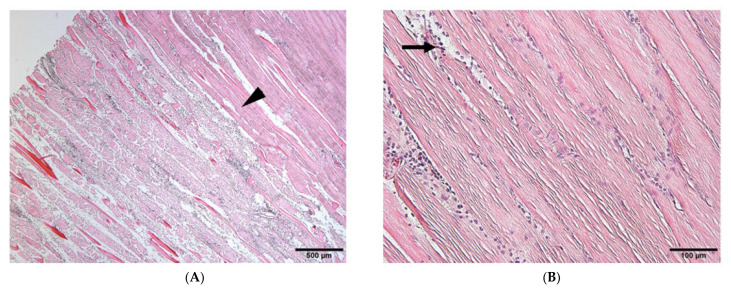
HE staining of the muscles 1 day after *bmp-2* transfer by needle- or plate-type electrodes. The damage area after *bmp-2* transfer by in vivo electroporation with plate-type electrodes (**B**) was wider than that with needle-type electrodes (arrow head). Inflammatory cells strongly stained with HE were more widespread in areas electroporated using needle-type electrodes than in those electroporated using plate-type electrodes (arrow) (**A**).

We also examined muscle sections harvested 1 day after electroporation using HE staining (Figure 5A,B). The area of muscle fiber damage caused by gene transfer by needle-type electrodes (Figure 5A, arrow head) was smaller than that caused by plate-type electrodes (Figure 5B, arrow head). Inflammatory cells strongly stained with HE were more widespread in areas electroporated using needle-type electrodes than in those electroporated using plate-type electrodes (Figure 5A,B, arrows).

## 4. Discussion

We successfully induced bone tissue formation in the targeted muscles using needle-type electrodes with a 100% success rate. In contrast, plate-type electrodes achieved only a 33% rate of inducing bone tissues. Our previous study revealed that a low voltage (<25 V) could not efficiently transfer a non-viral plasmid vector using plate-type electrodes without a skin incision, unlike the efficiency achieved with 100 V. However, when we performed a skin incision and directly applied plate-type electrodes on the muscles, we efficiently transferred a non-viral plasmid vector [39]. In this study, we performed gene transfer using plate-type electrodes without skin incision to reduce invasion to the target area. In contrast, needle-type electrodes were directly inserted into the targeted muscles, allowing for easier and more efficient tuning of electricity, which is known to affect the efficiency of gene transfer [39,40,41]. Although we need to consider other factors, such as the biocompatibility of electrode materials [42], needle-type electrodes may be suitable for gene transfer into skeletal muscles.

However, *bmp-4* gene-transferred muscles did not achieve a 100% success rate for osteoinduction with needle-type electrodes [43]. This may be derived from different types of BMP family members and different plasmid vectors. A comprehensive analysis of the osteogenic activity of 14 types of BMPs in osteoblastic progenitor cells suggested an osteogenic hierarchical model in which *bmp-2*, *-6*, and *-9*, not *bmp-4*, may play an important role in inducing the osteoblast differentiation of mesenchymal stem cells [44]. Moreover, a rat mandibular bone regeneration model required between 1 and 10 μg of *bmp-2* protein administration, whereas more than 10 μg of *bmp-4* protein administration was needed [45]. The pCAGGS plasmid vector has a high potential for gene expression in comparison with pMiw II, which is used for *bmp-4* gene expression [43] because it contains the cytomegalovirus immediate–early enhancer/chicken b-actin hybrid promoter [46]

Needle-type electrodes induced several smaller bone particles than plate-type electrodes in the targeted muscles. Needle-type electrodes require less electricity than plate-type electrodes (Figure 1C,D). The electric capacity of needle-type electrodes was represented in a two-dimensional area (Figure 1C), whereas that of plate-type electrodes could affect the three-dimensional area (Figure 1D). The electric field distribution might be an important factor in inducing uniformity over the plate compared with needle-type electrodes [47]. This may have contributed to the small particles of ectopic bone induced by needle-type electrodes in the target areas. However, some studies have reported that needle-type electrodes provide larger amounts of plasmid DNA in electroporation volumes than plate-type electrodes [48,49]. Therefore, *bmp-2* gene transfer to skeletal muscle with needle-type electrodes could be repeatedly performed. If small bone particles are separated, we can repeat gene transfer with needle-type electrodes to induce one-block bone formation after a clinical X-ray examination.

In future studies, we propose examining the time course of histological changes over an extended duration and measuring bone volume through three-dimensional and bone morphometric analyses. This will be carried out under various voltage conditions and with different needle electrode materials to assess biocompatibility.

## 5. Conclusions

*bmp-2* gene transfer with a non-viral plasmid vector and needle-type electrodes could induce bone tissue formation in skeletal muscles with a 100% success rate. Thus, this approach could be reliable and safe for bone grafting with skeletal muscles.

## Data Availability

Data are contained within the article.

## References

[1] Gomes-Ferrira P.H.S., Okamoto R., Ferreira S., De Oliveira D., Momessa G.A.C., Faverani L.P. (2016). Scientific evidence on the use of recombinant human bone morphogenetic protein-2 in oral and maxillofacial surgery. Oral Maxillofac. Surg..

[2] Burkus J.K., Gornet M.F., Dickman C.A., Zdeblick T.A. (2002). Anterior lumbar interbody fusion using rhBMP-2 with tapered interbody cages. J. Spinal Disord. Tech..

[3] Marx R.E., Harrell D.B. (2014). Translational research: The CD34+ cell is crucial for large-volume bone regeneration from the milieu of bone marrow progenitor cells in craniomandibular reconstruction. Int. J. Oral Maxillofac. Implant..

[4] Kim Y.J., Lee J.Y., Kim J.E., Park J.C., Shin S.W., Cho K.S. (2014). Ridge preservation using demineralized bone matrix gel with recombinant human bone morphogenetic protein-2 after tooth extraction: A randomized controlled clinical trial. J. Oral Maxillofac. Surg..

[5] Boyne P.J., Marx R.E., Nevins M., Triplett G., Lazaro E., Lilly L.C., Alder M., Nummikoski P. (1997). A feasibility study evaluating rhBMP-2/absorbable collagen sponge for maxillary sinus floor augmentation. Int. J. Periodontics Restor. Dent..

[6] Boyne P.J., Lilly L.C., Marx R.E., Moy P.K., Nevins M., Spagnoli D.B., Triplett R.G. (2005). De novo bone induction by recombinant human bone morphogenetic protein-2 (rhBMP-2) in maxillary sinus floor augmentation. J. Oral Maxillofac. Surg..

[7] Kao D.W., Kubota A., Nevins M., Fiorellini J.P. (2012). The negative effect of combining rhBMP-2 and Bio-Oss on bone formation for maxillary sinus augmentation. Int. J. Periodontics Restor. Dent..

[8] Sharan A., Madjar D. (2008). Maxillary sinus pneumatization following extractions: A radiographic study. Int. J. Oral Maxillofac. Implant..

[9] Bianchi J., Fiorellini J.P., Howell T.H., Sekler J., Curtin H., Nevins M.L., Friedland B. (2004). Measuring the efficacy of rhBMP-2 to regenerate bone: A radiographic study using a commercially available software program. Int. J. Periodontics Restor. Dent..

[10] Fiorellini J.P., Howel T.H., Cochran D., Malmquist J., Lilly L.C., Spagnoli D., Toljanic J., Jones A., Nevins M. (2005). Randomized study evaluating recombinant human bone morphogenetic protein-2 for extraction socket augmentation. J. Periodontol..

[11] Jensen E.D., Pham L., Billington C.J., Espe K., Carlson A.E., Westendorf J.J., Petryk A., Gopalakrishnan R., Mansky K. (2010). Bone morphogenic protein 2 directly enhances differentiation of murine osteoclast precursors. J. Cell. Biochem..

[12] Kawai M., Bessho K., Kaihara S., Sonobe J., Oda K., Iizuka T. (2003). Ectopic bone formation by human bone morphogenetic protein-2 gene transfer to skeletal muscle using transcutaneous electroporation. Hum. Gene Ther..

[13] Kawai M., Bessho K., Maruyama H., Miyazaki J., Yamamoto T. (2005). Human BMP-2 gene transfer using transcutaneous in vivo electroporation induced both intramembranous and endochondral ossification. Anat. Rec. Part A Discov. Mol. Cell. Evol. Biol..

[14] Kawai M., Bessho K., Maruyama H., Miyazaki J., Yamamoto T. (2006). Simultaneous gene transfer of bone morphogenetic protein (BMP)-2 and BMP-7 by in vivo electroporation induces rapid bone formation and BMP-4 expression. BMC Musculoskelet. Disord..

[15] Okubo Y., Bessho K., Fijimura K., Iizuka T., Miyatake S.I. (2000). Osteoinduction by bone morphogenetic protein-2 via adenoviral vector under transient. Biochem. Biophys. Res. Commun..

[16] Kaihara S., Bessho K., Okubo Y., Sonobe J., Kawai M., Iizuka T. (2004). Simple and effective osteoinductive gene therapy by local injection of a bone morphogenetic protein-2-expressing recombinant adenoviral vector and FK506 mixture in rats. Gene Ther..

[17] Simcikova M., Prather K.L., Praseres D.M., Monteiro G.A. (2015). Towards effective non-viral gene delivery vector. Biotechnol. Genet. Eng. Rev..

[18] Silvac I., Guay D., Mangion M., Champeil J., Gaillet B. (2017). Non-viral nucleic acid delivery methods. Expert Opin. Biol. Ther..

[19] Midoux P., Pigeon L., Gonqalves C., Pichon C. (2017). Peptides mediating DNA transport on microtubules and their impact on non-viral gene transfer efficiency. Biosci. Rep..

[20] Gantenbein B., Tang S., Guerrero J., Higuita-Castro N., Salazar-Puerta A.I., Croft A.S., Gazdhar A., Purmessur D. (2020). Non-viral gene delivery methods for bone and joints. Front. Bioeng. Biotechnol..

[21] Wang C., Pan C., Yong H., Wang F., Bo T., Zhao Y., Ma B., He W., Li M. (2023). Emerging non-viral vectors for gene delivery. J. Nanobiotechnol..

[22] Shchaslyvyi A.Y., Antonenko S.V., Tesliuk M.G., Teslegeev G.D. (2023). Current state of human gene therapy: Approved products and vectors. Pharmaceutials.

[23] Xiong R., Sauvage F., Fraire J.C., Huang C., De Smedt S.C., Braeckmans K. (2023). Photothermal nanomaterial-mediated photoporation. Acc. Chem. Res..

[24] Rosazza C., Meglic S.H., Zumbusch A., Rols M.-P., Miklavcic D. (2016). Gene electrotransfer: A mechanistic perspective. Gene Ther..

[25] Haberi S., Kanduser M., Flisar K., Hodzic D., Bregar V.B., Miklavcic D., Escoffre J.-M., Rols M.-P., Pavlin M. (2013). Effect of different parameters used for in vitro gene electrotransfer on gene expression efficiency, cell viability and visualization of plasmid DNA at the membrane level. J. Gene Med..

[26] Lambricht L., Lopes A., Kos S., Sersa G., Pre’at V., Vandermeulen G. (2016). Clinical potential of electroporation for gene therapy and DNA vaccine delivery. Expert Opin. Drug Deliv..

[27] Vandermeulen G., Vanvarenberg K., De Beuckelaer A., De Koker S., Lambrict L., Uyttenhove C., Reschner A., Vanderplasschen A., Grooten J., Pre’at V. (2015). The site of administration influences both the type and the magnitude of the immune response induced by DNA vaccine electroporation. Vaccine.

[28] Forjanič T., Miklavčič D. (2018). Numerical study of gene electrotransfer efficiency based on electroporation volume and electrophoretic movement of plasmid DNA. BioMed. Eng. OnLine.

[29] Gehl J. (2003). Electroporation: Theory and methods, perspectives for drug delivery, gene therapy and research. Acta Physiol. Scand..

[30] Mir L.M., Moller P.H., André F., Gehl J. (2005). Electric pulse-mediated gene delivery to various animal tissues. Adv. Genet..

[31] Pankaj K.M., Anuj G., Suresh C.G. (2014). Results of triple muscle (sartorius, tensor fascia latae and part of gluteus medius) pedicle bone grafting in neglected femoral neck fracture in physiologically active patients. Indian J. Orthopaed..

[32] Baksi D.D., Pal A.K., Baksi D.P. (2016). Osteosynthesis of ununited femoral neck fracture by internal fixation combined with iliac crest bone chips and muscle pedicle bone grafting. Indian J. Orthopaed..

[33] Betz O.B., Betz V.M., Abdulazim A., Penzkofer R., Schmitt B., Schroder C., Augat P., Jansson V., Muller P.E. (2009). Healing of large segmental bone defects induced by expedited bone morphogenetic protein-2 gene activated, syngeneic muscle grafts. Hum. Gene Ther..

[34] Betz O.B., Betz V.M., Schreader C., Penzkofer R., Goettlinger M., Wagner S., Augat P., Jansson V., Mueller P.E. (2013). Repair of large segmental bone defects: BMP-2 gene activated muscle grafts vs. autologous bone grafting. BMC Biotechnol..

[35] Deutinger M., Kuzbari R., Patemostro T., Todoroff B., Becker M.H. (1995). Functional and esthetic assessment of donor site defects following transfer of the gracilis muscle. Handchir. Mikrochir. Plast. Chir..

[36] Chen H.C., Sntamaria E., Chen H.H., Cheng M.H., Tang Y.B. (1999). Microvascular vastus lateralis muscle flap for chronic empyema associated with a large cavity. Ann. Thorac. Surg..

[37] Schneider C.A., Rasband W.S., Eliceiri K.W. (2012). NIH Image to ImageJ: 25 Years of image analysis. Nat. Methods.

[38] Zhang X., Qu Q., Yang A., Wang J., Cheng W., Deng Y., Zhou A., Lu T., Xiong R., Huang C. (2023). Chitosan enhanced the stability and antibiofilm activity of self-propelled Prussian blue micromotor. Carbohydr. Polym..

[39] Yamamoto H., Kawai M., Shiotsu N., Watanabe M., Yoshida Y., Suzuki K., Maruyama H., Miyazaki J., Ikegame M., Bessho K. (2012). BMP-2 gene transfer under various conditions with in vivo electroporation and bone induction. J. Oral Maxillofac. Surg. Med. Pathol..

[40] Taylar J., Babbs C.F., Alzghoul M.B., Olsen A., Latour M., Pond A.L., Hannon K. (2004). Optimization of ectopic gene expression in skeletal muscle through DNA transfer by electroporation. BMC Biotechnol..

[41] Mir L.M. (2008). Application of electroporation gene therapy: Past, current, and future. Methods Mol. Biol..

[42] Li Y., Wang J., Satterle A., Wu Q., Wang J., Liu F. (2012). Gene transfer to skeletal muscle by site-specific delivery of electroporation and ultrasound. Biochem. Biophys. Res. Commun..

[43] Kishimoto K.N., Watanabe H., Nakamura H., Kokubun S. (2002). Ectopic bone formation by electroporatic transfer of bone morphogenetic protein-4 gene. Bone.

[44] Cheng H., Jiang W., Phillips F.M., Haydon R.C., Peng Y., Zhou L., Luu H.H., An N., Benjamin B., Vanichakarn P. (2003). Osteogenic activity of the fourteen types of human bone morphogenetic proteins (BMPs). J. Bone Jt. Surg. Am..

[45] Arosarena O., Collins W. (2005). Comparison of BMP-2 and -4 for rat mandibular bone regeneration at various doses. Orthod. Craniofac. Res..

[46] Niwa H., Yamamura K., Miyazaki J. (1991). Efficient selection for high-expression transfectants with a novel eukaryotic vector. Gene.

[47] Gehl J., Sørensen T.H., Nielsen K., Raskmark P., Nielsen S.L., Skovsgaard T., Mir L.M. (1999). In vivo electroporation of skeletal muscle: Threshold, efficacy and relation to electric field distribution. Biochim. Biophys. Acta (BBA) Gen. Subj..

[48] Gothelf A., Gehl J. (2010). Gene electrotransfer to skin; review of existing literature and clinical perspectives. Curr. Gene Ther..

[49] Gothelf A., Mahmood F., Dagnaes-Hansen F., Gehl J. (2011). Efficacy of transgene expression in porcine skin as a function of electrode choice. Bioelectrochemistry.

